# Richness and density jointly determine context dependence in bacterial interactions

**DOI:** 10.1016/j.isci.2023.108654

**Published:** 2023-12-07

**Authors:** Keven D. Dooley, Joy Bergelson

**Affiliations:** 1 Committee on Microbiology, University of Chicago, Chicago, IL 60637, USA; 2 Center for Genomics and System Biology, Department of Biology, New York University, New York, NY 10003, USA

**Keywords:** Microbiology, Bacteriology, Synthetic biology

## Abstract

Pairwise interactions are often used to predict features of complex microbial communities due to the challenge of measuring multi-species interactions in high dimensional contexts. This assumes that interactions are unaffected by community context. Here, we used synthetic bacterial communities to investigate that assumption by observing how interactions varied across contexts. Interactions were most often weakly negative and showed a phylogenetic signal among genera. Community richness and total density emerged as strong predictors of interaction strength and contributed to an attenuation of interactions as richness increased. Population level and per-capita measures of interactions both displayed such attenuation, suggesting factors beyond systematic changes in population size were involved; namely, changes to the interactions themselves. Nevertheless, pairwise interactions retained some explanatory power across contexts, provided those contexts were not substantially divergent in richness. These results suggest that understanding the emergent properties of microbial interactions can improve our ability to predict the features of microbial communities.

## Highlights

• Most interactions were weakly negative and showed a phylogenetic signal among genera
• Interactions were context-dependent and attenuated in more complex communities
• Community richness and total density were predictors of interaction strength

## Graphical abstract

**Figure:**
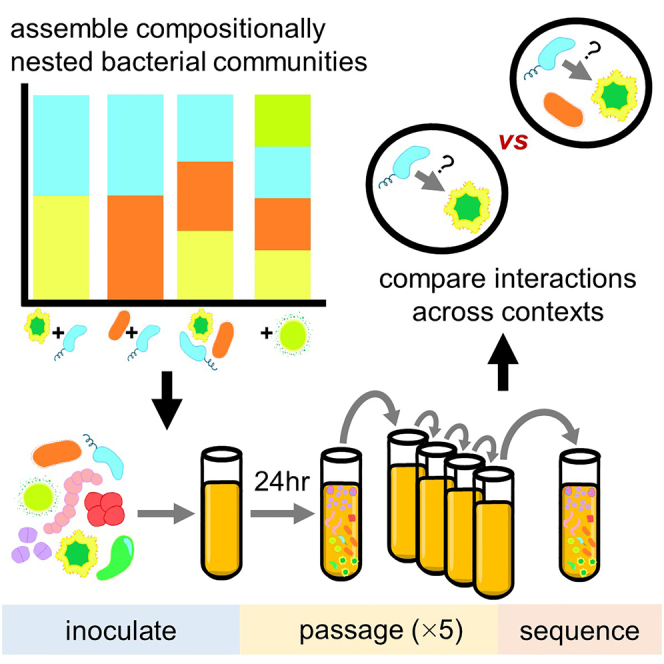

## Introduction

Microbes are the engines of many biochemical processes that support life on Earth.^1^ Importantly, however, microbes rarely perform these complex functions in isolation, instead acting within communities. Many efforts are thus underway to design microbial communities that perform desired functions, enabling us to co-opt these powers of chemical transformation and develop applications relevant to human health, agriculture, and industry.^2^^,^^3^ However, the intricate relationships underlying such complex functions provide a challenge that must be overcome, as interactions among members constrain the extent to which the abundance and distribution of a focal microbe can be manipulated. Overcoming this challenge will require an understanding of the forces that determine the structure and function of microbial communities.

Interactions between community members have long been known to affect community composition^4^^,^^5^^,^^6^ and therefore the emergent functions performed by a community.^7^^,^^8^ Leveraging an understanding of interspecific interactions is a promising and actively researched approach for designing the structure and function of microbial communities.^9^^,^^10^ However, for such an approach to be effective, observations of interactions made in one community context must inform the extent of that interaction in another context.

Interactions are often modeled as a network of static pairwise per-capita or proportional effects between members of a community.^11^^,^^12^^,^^13^ By assuming that it is appropriate to distill an interaction into a simple static relationship, we can reduce the complexity of interaction networks^14^ and apply knowledge of interactions gleaned from other contexts to make predictions about unobserved communities.^15^ However, a variety of known effects call this simplification into question. Interactions can be subject to higher order effects (“higher order interactions” or “HOIs”) where a pairwise interaction is altered by the presence of one or more other community members.^16^^,^^17^^,^^18^ Habitat modification can also affect microbial interactions,^19^ an example being environmental pH modification, which has been observed as a relevant factor in microbial community assembly.^20^^,^^21^^,^^22^ Due to effects such as these, knowledge of pairwise interaction strength or coexistence can have limited predictive power in complex communities.^23^^,^^24^ Thus, advancing our understanding of what contributes to the variation of interactions between contexts stands to facilitate the rational design of microbial communities.

One potential solution to these complexities is to identify patterns in how pairwise interactions vary across contexts and uncover the underlying drivers of this variation. Such an understanding stands to improve our predictions of how microbial interactions will change between community contexts. Encouragingly, recent work has demonstrated that stronger negative interactions are found at high nutrient concentrations,^22^ confirming the possibility of identifying broadly general patterns. By expanding our understanding of such patterns, we hope to improve the predictive power of pairwise interactions. Here, we use synthetic bacterial communities to observe how interactions vary across community contexts and identify patterns underlying that variation.

## Results

### Assembly of synthetic communities

**Figure 1:**
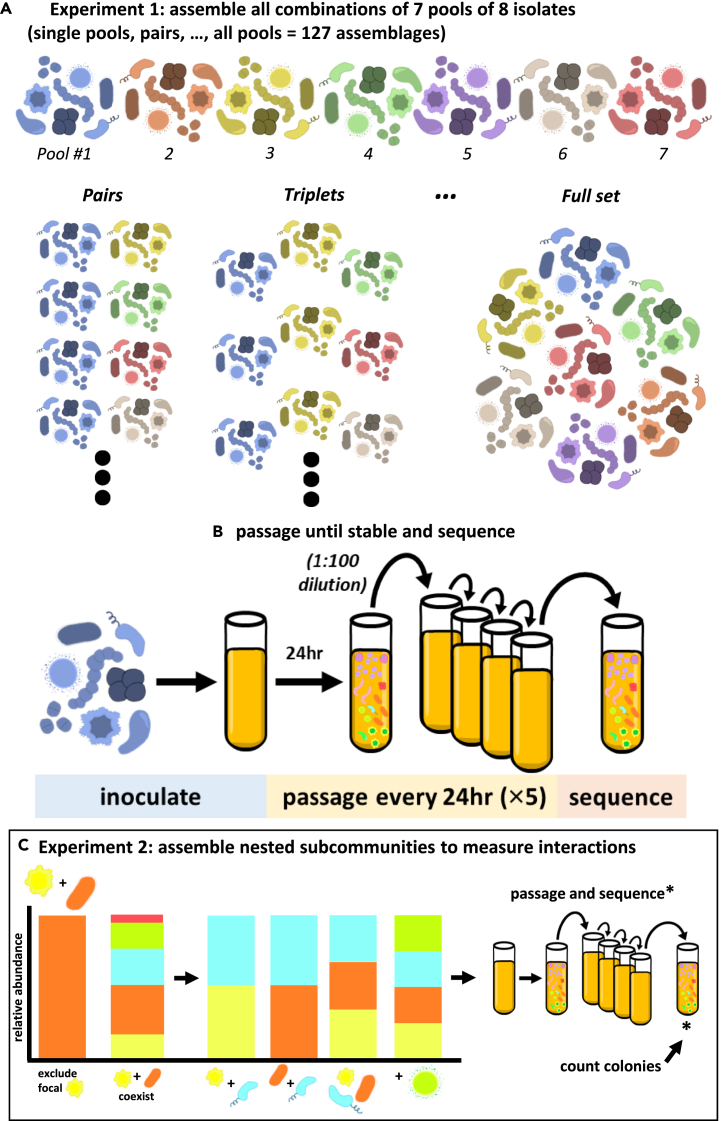
Experimental outline (A) A set of 56 isolates representing 21 genera were randomly pooled into 7 pools. All combinations of those pools were assembled at equal titers, with respective densities scaled to the total number of isolates initially present. (B) These combinations were inoculated in triplicate into a custom medium derived from *Arabidopsis* leaves (ALM) and passaged daily into fresh medium at a 1:100 dilution for 5 days. To characterize the community compositions, the day-6 samples were sequenced, and short reads were mapped to reference genomes. (C) Ten communities displaying context-dependent coexistence were decomposed into nested subcommunities containing the focal isolate and/or putative excluder isolate. These communities were assembled, passaged, and sequenced as described for the previous communities. To provide the absolute abundance information necessary to measure interactions, the final timepoint (day 6) was quantified by counting colonies on 1× TSA plates.

We assembled a set of synthetic communities from a pool of 56 bacterial strains isolated from the leaves of wild and field-grown *Arabidopsis thaliana* by randomly dividing isolates into seven pools of eight members. We then created 127 unique communities representing all possible combinations of those pools (i.e., seven single pool communities, twenty-one two pool communities, and so forth) (Figure 1A). These communities were inoculated into a custom growth medium derived from *A. thaliana* leaves (*Arabidopsis* leaf medium, ALM) (STAR Methods) at a consistent total community titer, with each member accounting for an equal proportion of the population given the initial richness (number of community members). To allow the communities to reach a steady state reflective of their long-term composition, we passaged each community for 6 days by performing a 1:100 dilution into fresh medium every 24 h (Figure 1B). This period was sufficiently long to allow the community composition to stabilize (Figure S1). We characterized the compositions of these final communities by mapping Illumina short reads against a nearly complete and high-quality genome of each isolate (STAR Methods).

### Measurement of interactions

We screened this set of 127 communities for putative interactions by finding pairs of communities where a focal isolate was observed to coexist alongside one or more specific isolates in one community context but was excluded in another context (Figures 1C and S2). We posited that such context-dependent coexistence was related to interactions between the focal isolate and/or its context-dependent excluder with additional members of the community. Thus, from all paired communities in which we observed context-dependent coexistence, we selected a set of ten pairs that maximized compositional diversity in which to investigate potential interactions.

**Figure 2:**
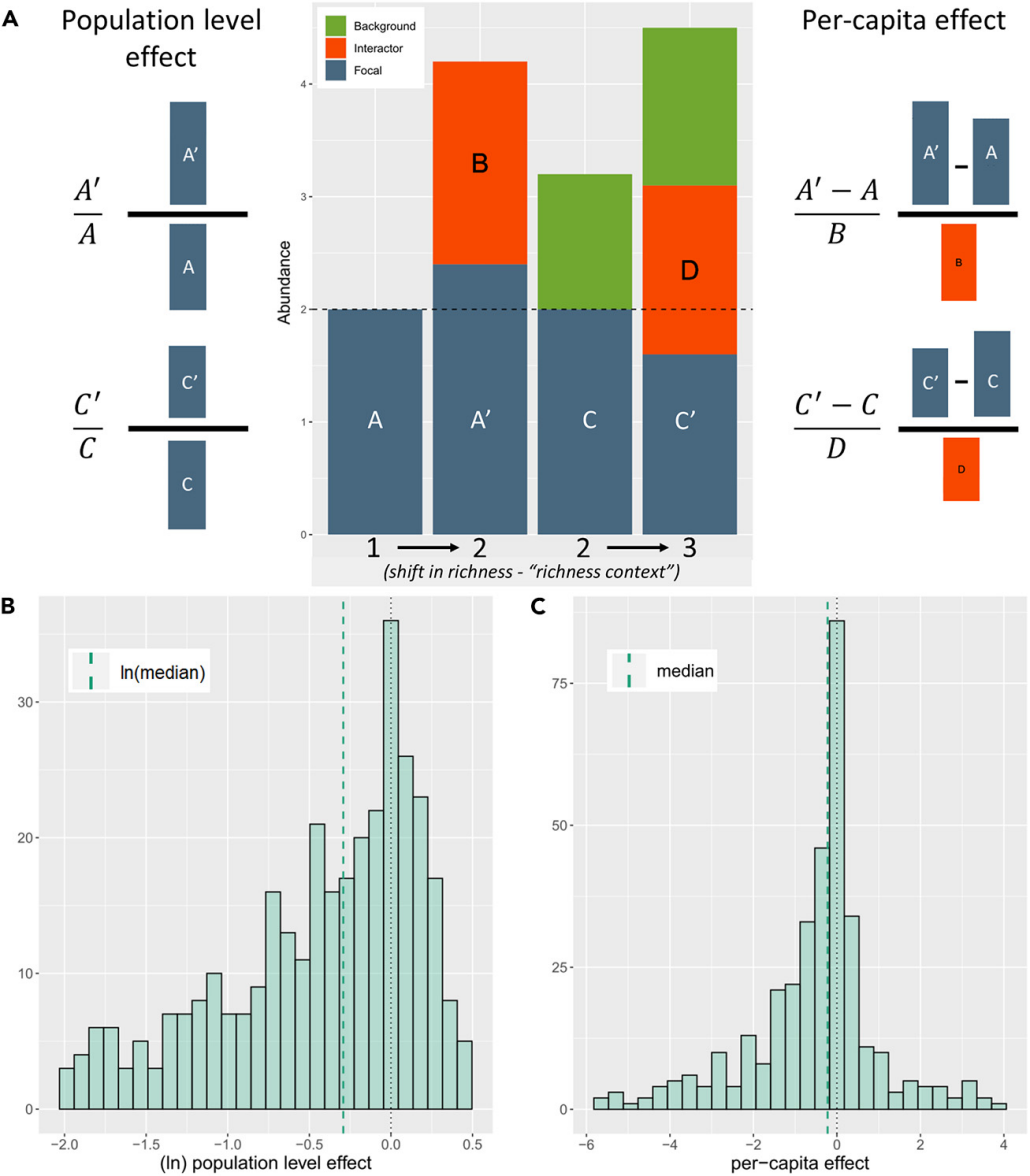
Distributions of observed interactions (A) Interactions between a “focal” isolate and “interactor” isolate were calculated as two measures, 1) a population effect, calculated as the ratio of the focal isolate’s density with and without the interactor present, and 2) a per-capita effect, calculated as the change in density of the focal isolate between contexts with and without the interactor, scaled by the abundance of the interactor. Interactions were always calculated between communities, varying by a single isolate – the interactor. However, additional isolates (“background” isolates) could also be present in the compared communities. The “richness context” of an interaction refers to the richness of the pairs of community contexts from which an interaction is observed (e.g., 1=>2 for the first example interaction, 2=>3 for the second example interaction including a “background” isolate). (B) The distribution of all observed interactions, as population level effects, (natural-log transformed to symmetrize ratios). (C) The distribution of all observed interactions, as per-capita effects. In B and C, the dotted black line marks 0, and the dashed green line marks the median respective interaction (natural-log transformed for the population level effects).

To do so, we decomposed these communities into (non-exhaustive) sets of nested subcommunities varying by a single isolate (Figure 1C), and always including a focal isolate and/or its excluder. In total, we assembled 245 such communities, ranging in initial richness from 2 to 8 isolates, and passaged them for 6 days, as previously described. A subset of communities was passaged for 12 days, with samples from days 6 and 12 sequenced to confirm that community composition was stable by day 6 (Figure S3). With these sets of nested communities, we were able to measure the effect of one isolate on the abundance of another (i.e., an interaction) across multiple community contexts (Figure 2A).

Importantly, interactions are measured as changes in absolute abundance, but characterizations of community composition obtained through sequencing are limited to relative abundance information. Thus, we measured absolute abundances by counting colonies from serial dilutions of each day-6 sample, which we then used to translate the relative abundances obtained from sequencing into absolute abundances. We chose to estimate absolute abundances by counting colonies, as it is inexpensive, relatively high-throughput, and a more direct measure of abundance than optical density. However, it is still subject to its own biases, namely, differences in plating viability between bacterial isolates. To reduce this effect, we plated on a rich medium on which all isolates displayed robust growth and counted colonies over the course of a week to ensure that isolates slow to grow in the plated environment were counted (STAR Methods).

With measures of absolute abundance in hand, we measured interactions by comparing abundances between pairs of communities that varied by a single member (the “interactor"). For example, the interaction between a “focal” isolate A and an “interactor” isolate B was observed by comparing the abundance of A in a community lacking B to the abundance of A in a community where B was present. Here, we measure interactions using two metrics (Figure 2A and STAR Methods) and refer to the signed effect of an interaction as its “strength” and the absolute value as the “absolute strength.” Our first metric measures an interaction as the ratio of the focal isolate’s abundance in the context with the interactor to its abundance in the context without the interactor. This is a commonly used metric,^25^^,^^26^^,^^27^^,^^28^^,^^29^ which represents an interaction as a population level effect on the focal isolate. Our second metric measures an interaction as the per-capita effect of an interactor on the abundance of a focal isolate.^14^ The population level effect of an interaction is a function of the per-capita effect of an interactor scaled by the density of that interactor in a given community context. This is relevant, as later in discussion we will show that a general relationship between richness and density existed in our communities and contributed to the observed effect of interactions.

### Negative interactions were more common and stronger than positive interactions

Coexistence was common among the 245 subcommunities (exclusion of one or more isolates occurred in only 28% of communities), thus we observed many compositionally nested communities from which we could measure interactions. We observed a total of 388 pairwise interactions across all community contexts (Figure 2). Negative interactions were more common, representing 67% of interactions. We observed median values of −0.25 for the population level and −0.27 for the per-capita effects, respectively. Negative interactions (n = 262) were statistically greater in absolute strength than positive interactions (n = 126) for both measures (Figure S4A, Wilcoxon rank-sum test: p values <4e^−7^ and 0.003, respectively). As a note, non-parametric tests were chosen due to the nonnormal distribution of the observed interactions. We also observed support for phylogenetic effects among genera at both the population level and per-capita measures of interactions. Namely, interactions between isolates belonging to the same genus (n = 56) tended to be more negative than those belonging to distinct genera (n = 332) (Figure S4B, Wilcoxon rank-sum test: p values 0.008 and 0.039, population and per-capita, respectively).

### Individual interactions attenuated as richness increased

**Figure 3:**
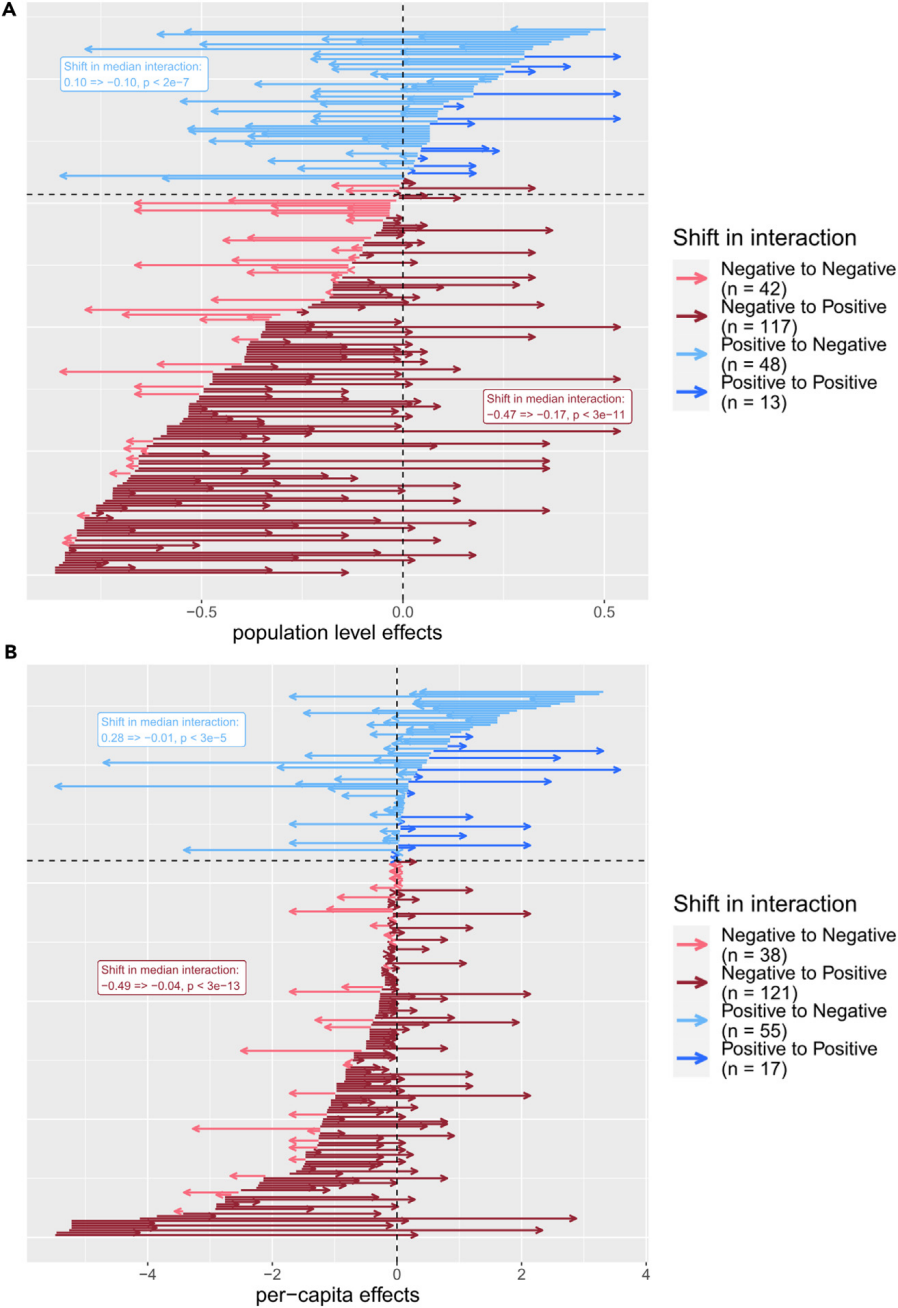
Interactions attenuated as richness increased The shift in interactions between richness contexts varying by a richness of one for A) population level (presented as “ratio – 1”), and B) per-capita effects. All comparisons of an interaction were compared between richness contexts varying in richness by a single isolate. Each arrow on the plots represents an interaction observed in two separate richness contexts (e.g., 1=>2 and 2=>3 community members), with the tail of the arrow representing the value of the interaction in the lower richness context and the head of the arrow representing the value observed in the higher richness context (population level: n = 220, per-capita: n = 231). Arrows are colored by the initial direction and the shift in direction of an interaction. For ease of interpretation, interactions were ordered along the y axis by initial interaction strength. Some interactions were observed in multiple higher richness contexts and are thus presented as multiple arrows with tails aligned on the x axis. Labels display the shift in median interactions for initially positive and negative interactions, respectively, with p values summarizing the outcome of Wilcoxon signed-rank tests to determine if the shift in interaction value represented a significant change.

Our method of measuring interactions was to compare contexts that differ in composition by a single “interactor” isolate. To observe how interactions changed between contexts, we compared the strength of pairwise interactions measured in community contexts with or without a single “background” isolate. In this way, we compared interactions between two pairs of communities that differed in “richness context” by one (Figure 2A). As an example, we might compare an interaction measured when only the focal and interactor are present (richness context 1=>2) with that interaction measured when a single “background” isolate was also present in both contexts (richness context 2=>3). The use of our complete dataset enabled us to analyze a total of 272 instances of such paired contexts. First focusing on the full set of population level effects, we observed that interactions generally attenuated in absolute strength when measured in a community with one additional background member (median difference −0.1, paired Wilcoxon signed rank test: n = 220, p value 0.008). When grouping interactions by their initial direction, the median positive and negative interaction became less positive and negative, respectively (Figure 3A). However, when considering the absolute strength of initially negative (n = 159) or positive interactions (n = 61), the interaction effect was significantly weaker for initially negative interactions but significantly stronger for initially positive interactions (paired Wilcoxon rank-sum test: p values < 2e^−7^ and 0.011, negative and positive, respectively). This result for the initially positive interactions manifests as a shift from a weakly positive median interaction to a moderately negative median interaction.

As previously stated, the population level effect of an interaction is a function of the per-capita effect of an interactor and the density of that interactor in each community context. Thus, the observed decrease in population level effects suggests a decrease in the strength of per-capita effects and/or a systematic decrease in interactor density. Indeed, there was a positive relationship between absolute population level effects and interactor abundance (Pearson’s r = 0.24, n = 388, p value < 2e^−6^). Further, when we consider how individual per-capita interactions shifted across contexts, we observe again that absolute interaction strength attenuated as richness context increased (median difference −0.15, paired Wilcoxon signed rank test: n = 231, p value 0.002). As with the population level effects, per-capita effects grouped by initial direction showed consistent shifts (Figure 3B). When considering absolute strength of initially negative (n = 159) or positive interactions (n = 72), absolute strength became significantly weaker for initially negative interactions (paired Wilcoxon rank-sum test: p value 0.0001); however, unlike at the population level, it remained statistically unchanged for positive interactions (paired Wilcoxon rank-sum test: p value 0.89). These results suggest that part of the decrease in the population level effects can be explained by a decrease in the per-capita effects. Next, we evaluate an alternative explanation by investigating the relationships between richness and density in our communities.

### Relationships between richness and density help explain trends in population effects across richness

**Figure 4:**
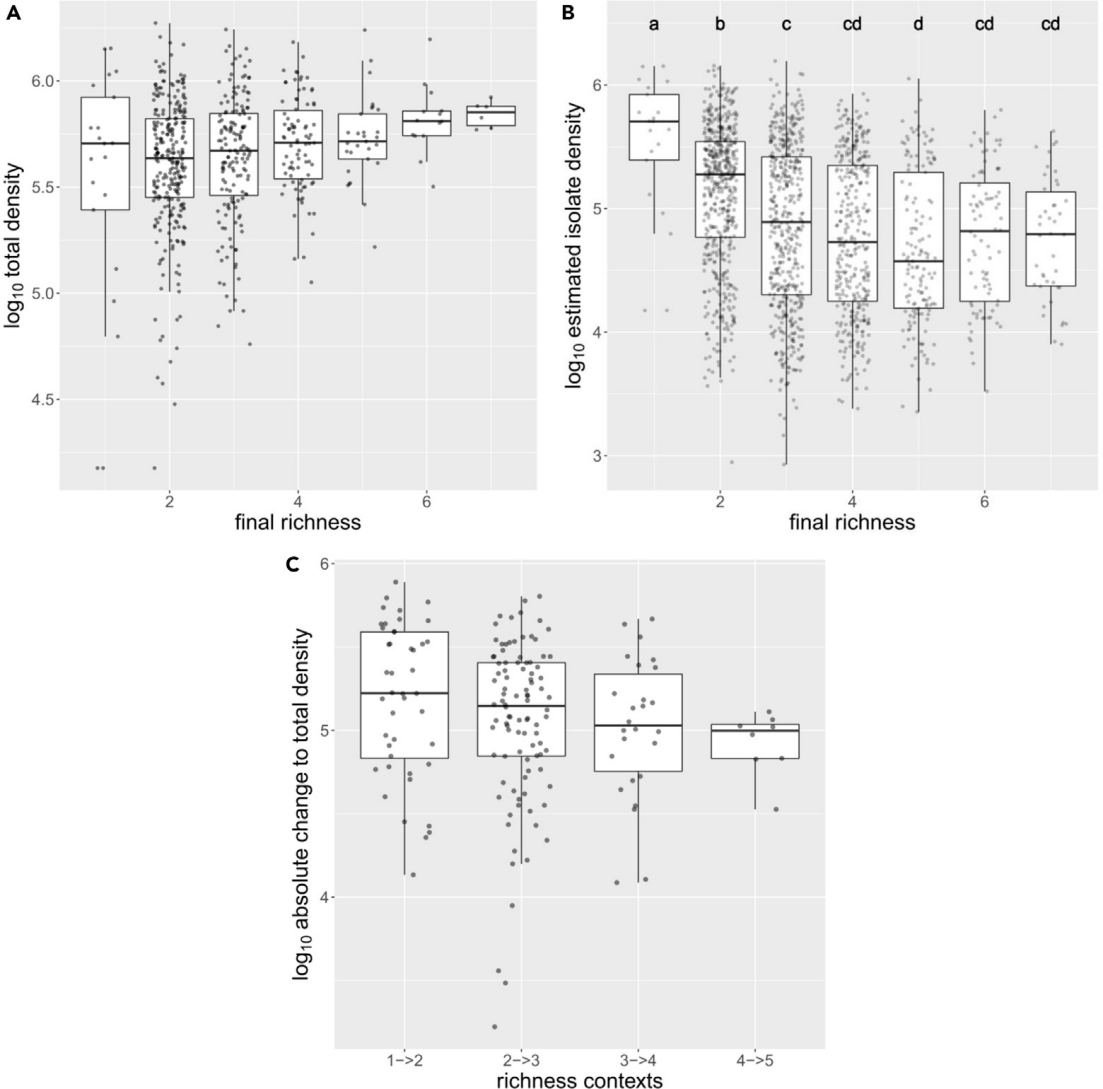
Relationships between richness and density (A) the relationship between final community richness and total community density, (B) the relationship between richness and individual isolate density (significantly distinct groups were determined through post hoc pairwise t-tests using the Holm method for multiple testing correction), (C) the absolute change in total community density associated with an interaction, grouped by the richness context in which interactions were observed (interactions from richness contexts with fewer than 3 observations were removed). For all plots, density is plotted on a log scale.

We observed that, as richness increased, the average total density of communities gradually increased to a modest extent (Figure 4A, Pearson’s r: 0.18, n = 577, p value <2e^−5^), while the average density of each member decreased before reaching an asymptote in communities with four members (Figure 4B). Further, all isolates that demonstrated a significant relationship between richness and individual density displayed negative relationships (Figure S5). This general decrease in density with increasing richness helps explain the observed attenuation of interactions when measured as a population effect. Namely, because individual densities decreased with an increase in community richness, the population level effect of the interactor should decrease. Indeed, interactor density explained a significant portion of the variance in population level effects (linear regression: adjusted R^2^ = 0.1, n = 336, p value <3e^−9^). Additionally, the relationship between the densities of community members and total community density meant that as richness increased, the absolute change in total density associated with an interaction decreased (Figure 4C, Pearson’s r: −0.23, n = 177, p value 0.002). In other words, adding a given interactor to a community resulted, on average, in a smaller change to total community density in higher richness contexts.

### Interactions have explanatory power between contexts

We next asked, how well does the effect of an interaction observed in one community context describe its effect in another context? We attempted to answer this question by modeling the effect of an interaction in one context, informed by that interaction observed in a different context and/or other community properties associated with the focal context.

First, we identified community properties associated with the interaction effect, specifically focusing on richness and change in total density. While modeling the interaction effect (i.e., change in focal isolate abundance) associated with all 388 observed interactions, both richness and the change in total density emerged as highly explanatory variables (Table 1). The change in total density was more explanatory with an adjusted R^2^ value of 0.33 compared to 0.08 for richness. A joint model including both variables and their interactions could explain 57% of the variance in the interaction effect. We also note that the explanatory power of total density grouped by richness context (1=>2, 2=>3, and so forth) decreased in higher richness contexts, but remained a significant predictor for all richness contexts other than “6=>7” (Table S2).

**Table 1:** Summary of linear regressions modeling the effect of an interaction on emergent community properties

Model (n *= 388 interactions*)	df	adjusted R^2^	p value
focal change ∼ total change	1	0.334	<2.2e^−16^
focal change ∼ n-context	5	0.083	2.2e^−7^
focal change ∼ total change ∗ n-context	11	0.565	<2.2e^−16^

“Focal change” indicates the change in density of the focal isolate in the predicted context. “Total change” indicates the change in total density between the community contexts of the interaction. “n-context” indicates the richness contexts over which the interaction was observed (as a factor). An “∗” in the model indicates an interaction term in addition to the separate effects. We modeled all 388 observed interactions. The “p value” column indicates the p value of the model itself.

Next, we used the set of 272 paired interaction contexts differing by a richness of one to evaluate if the effect of an interaction observed in one context was informative in describing the effect in another context (e.g., compare interaction effects between richness contexts 1=>2 and 2=>3). In this dataset, interactions in one context were able to describe ∼16% of the variance in the change in density of a focal isolate (i.e., interaction effect) in the other context (Table 2). A model using the change in the total density of the focal context (identified above as strongly associated with interaction effect) explained ∼27% of the variance, while the richness context only explained ∼3%. Given the explanatory power of the change in total density, we wanted to evaluate if this variable was statistically distinct from the interaction effect across contexts. Thus, we evaluated a joint model of these two variables (change in total abundance of the focal context and interaction effect in another context) and found that it explained ∼42%, suggesting that the two variables are largely independent. However, expanding the dataset to consider comparisons between any observations of a given interaction (e.g., compare richness contexts 1=>2 and 4=>5) reduced the explanatory power of interactions between contexts to ∼10% of variance, suggesting the consistency of interactions decays as communities diverge in richness and composition (Table S3). Ultimately, these results demonstrate the persistent but limited explanatory power of interactions across contexts and highlight the relevance of community level properties in understanding the assembly of microbial communities.

**Table 2:** Summary of linear regressions modeling the explanatory power of interactions between contexts differing in richness context by a single community member

Model (n *= 272 paired interactions*)	df	adjusted R^2^	p value
focal change ∼ total change	1	0.273	<2.2e^−16^
focal change ∼ n-contexts	4	0.026	0.024
focal change ∼ interaction effect	1	0.159	<2e^−12^
focal change ∼ total change + interaction effect	2	0.417	<2.2e^−16^

“Focal change” indicates the change in density of the focal isolate in the predicted context. “Total change” indicates the change in total density between the community contexts of the interaction. “n-contexts” indicates the richness contexts from the pair of interactions (e.g., 1=>2 & 2=>3). “Interaction effect” indicates the change in density of the focal isolate in the interaction context which was not being predicted. We modeled all 272 interactions that were observed between richness contexts differing by a single community member. We considered the explanatory power of interactions from the bottom-up, i.e., “interaction effects” came from the lower richness context (continuing the example above, 1=>2), while “total change” came from the predicted higher richness context (2=>3), as in the models described in Table 1. A “+” in the model indicates the separate effects with no interaction term. The “p value” column indicates the p value of the model itself.

## Discussion

Here, we used synthetic bacterial communities to observe a large set of interactions across community contexts, ranging from the simplest possible community of two coexisting isolates to complex communities with up to seven isolates. These interactions were, on average, weakly negative and displayed a phylogenetic effect, in alignment with other studies of microbial interactions.^27^^,^^28^^,^^30^ However, positive interactions were not uncommon, an observation that has gained growing recent empirical support.^25^^,^^27^ When comparing interactions across contexts of increasing richness, we observed a general attenuation of interactions, though this arose predominately due to a consistent shift in negative interactions (Figure 3). We observed that much of this change can be explained by relationships between individual density and richness/total density (Table 1 and Figure 4). Namely, as richness increased, the modest increase in total density resulted in a decrease in individual isolate density (Figure 4B). This relationship can help explain the observed attenuation of population level effects, as decreased density of interactor isolates in higher richness contexts should lead to smaller effects and did, in fact, explain ∼9% of variance in population level effects. However, the per-capita effects also showed some decrease in strength with an increase in richness, at least for negative interactions, suggesting additional processes were present that imparted a systematic change in the interactions.

Why would per capita effects be attenuated at high richness, and why predominately among initially negative interactions? Previous observation of the attenuation of pairwise interactions in the zebrafish gut was attributed to the effect of higher order interactions,^31^ though that study was unable to identify the mechanisms of such effects. We have a similarly limited mechanistic understanding of observed interactions and what underpins their variation between community contexts. The importance of HOIs in microbial community assembly remains an actively debated subject, with theoretical and empirical evidence to support both sides.^7^^,^^18^^,^^32^^,^^33^^,^^34^^,^^35^^,^^36^ However, HOIs are challenging to appropriately identify,^16^^,^^37^^,^^38^ and our lack of fully characterized interaction networks precludes us from determining their relevance here.

Another possible explanation for the attenuation of per-capita effects is non-additivity in interactions. In other words, overlap in the mechanisms underpinning how multiple interactors affect a given focal isolate could result in a reduced per-capita effect when multiple interactors are present. Such non-additivity has been recently reported.^25^ This effect would be likely if metabolic interaction (such as competition over labile carbon sources) predominately underlies interactions and community assembly, as has been shown in synthetic communities that were organized into functional guilds by preferred metabolic strategy.^39^^,^^40^^,^^41^ In the context of how we measure and compare interactions here, such mechanistic overlap would be hypothesized to reduce the impact of a novel interactor due to a function already being performed by a “background” isolate in the community. Such mechanistic redundancy would be probabilistically more likely as richness increases.

Despite the limitations of our data, some insights can be inferred by asking what gives rise to the relationships we observed between richness and individual or total density. The apparent modest increase in total density in higher richness communities might have emerged for two reasons: 1) larger initial pools of isolates entailed greater metabolic diversity, thus allowing the community to occupy more of the available niche space, or 2) larger initial pools may simply have had a greater chance of including one or more isolates with high fitness in the environment (a “sampling effect”).^42^ Both possibilities would result in higher levels of community metabolic activity at higher levels of richness, which has been observed to have a positive effect on those community members with relatively low fitness as a result of cross-feeding or general metabolic leakiness.^27^^,^^43^^,^^44^ In this way, positive effects absent in simpler contexts may have emerged in more complex settings. This hypothesis would address the fact that we predominately observed attenuation among negative interactions, as it would result in an apparent decrease in the per-capita effect while actually representing an independent emergent positive effect.

A key finding here was that the relationship between individual isolate density and richness/total community density was informative for predicting the change in abundance of an isolate between community contexts (Table 1). But why were changes in total density informative of changes in individual density? We suggest that this result arose because individual isolate density decreased as richness increased (Figure 4B) due to the modest changes in total density (Figure 4A). The associated attenuation of interactions in higher richness contexts was inherently observed as a decreased change in individual density but also a decreased change in total community density (Figure 4C). This link between the two effects meant that the change in total density was an informative predictor of the change in individual density (i.e., interaction effect) as well. Nonetheless, interaction effects themselves were useful predictors across contexts (Table 2), suggesting that context-dependency generally does not redefine an interaction, but instead changes interactions to varying degrees. Indeed, we observed that the explanatory power of interactions decayed as the divergence between community contexts increased (Table S3). Such an outcome is in line with results from other studies, as it has been shown that predictions of coexistence based on pairwise cultures decay as the complexity of the predicted community increases.^23^

We sought to advance our understanding of microbial interactions by observing how they vary across contexts and identifying patterns in that variation. Our observation of the general attenuation of interactions as richness increased is a straightforward and potentially useful result. And our finding that the relationships between individual density, richness, and total density could help explain changes in pairwise interactions demonstrates both the usefulness of understanding community level properties and the value of considering interactions from the per-capita perspective. The observation that negative per-capita interactions nonetheless generally attenuate with richness suggests that context-dependency of interactions is a common feature in microbial communities. Further study of the specific processes that give rise to such context dependence would be a fruitful endeavor that, combined with the observed population level processes, may improve our ability to predict the structure and function of microbial communities.

## STAR★Methods

### Key resources table

**Table:** 

REAGENT or RESOURCE	SOURCE	IDENTIFIER
**Critical commercial assays**		

Quant-iT PicoGreen dsDNA Assay Kit	Invitrogen	Catalogue #: P7589
Blue Pippin DNA Size Selection (2% agarose gel cassette)	Sage Science	Product #: BDF2010
TapeStation D1000 ScreenTape Assay	Agilent	Part #: 5067-5582
KAPA Library Quantification Kit	Roche	Catalogue #: 07960140001

**Experimental models: Organisms/strains**		

Arabidopsis thaliana: KBS-Mac-74	Bergelson Laboratory	ABRC stock number: CS78969

**Deposited data**		

Bacterial genomes and experimental sequencing data	This paper	GenBank Accession PRJNA953780: https://www.ncbi.nlm.nih.gov/bioproject/PRJNA953780/

**Software and algorithms**		

R	Publicly available	https://www.r-project.org/
Anvi’o	Publicly available	https://anvio.org/
Spades	Publicly available	https://cab.spbu.ru/software/spades/
BBtools	Publicly available	https://jgi.doe.gov/data-and-tools/software-tools/bbtools/

### Resource availability

#### Lead contact

Keven D. Dooley, kevendooley@gmail.com.

#### Materials availability

Bacterial genomes and experimental sequencing data generated in this study have been deposited to GenBank (Accession PRJNA953780).

#### Data and code availability

• All data reported in this paper will be shared by the lead contact upon request.
• All original code is available in this paper’s supplemental information.
• Any additional information required to reanalyze the data reported in this paper is available from the lead contact upon request.

### Experimental model and subject details

#### Bacterial strains

All bacterial strains were originally isolated from the leaves or roots of wild or field grown *Arabidopsis thaliana* in the midwestern states of the USA, specifically: IL, IN, MI (Methods S2). Strains were cultured at 28°C in a custom leaf-based culturing medium, “*Arabidopsis* leaf medium”.

#### Arabidopsis leaf medium (ALM)

*Arabidopsis thaliana* (KBS-Mac-74, accession 1741) plants were grown in the University of Chicago greenhouse in sterile potting soil at 50% humidity from January to March 2020. Seeds were densely planted in 15-cell planting trays, stratified for 3 days in the dark at 4°C, then moved to the greenhouse and thinned after germination to 4-5 plants per cell. Above ground plant material was harvested just before development of inflorescence stems. Plant material was coarsely shredded by hand before adding 100g to 400mL of 10mM MgSO_4_ and autoclaving for 55 minutes. After cooling to room temperature, the medium was filtered through 0.2μm polyethersulfone membrane filters to maintain sterility and remove plant material. The medium was stored in the dark at 4°C. Before being used for culturing, the medium was diluted 1:10 in 10mM MgSO_4_.

### Method details

#### Experimental set up and culturing

Fresh bacterial stocks were prepared by first inoculating the isolates into 1mL of ALM shaking at 28°C and growing overnight. 100μL of these cultures were then used to inoculate 5mL of ALM shaking at 28°C. Once the cultures were visibly turbid, they were divided into 1mL aliquots with sterile DMSO added to a final concentration of 7% as a cryoprotectant. Stocks were stored at -80°C. Additionally at this time, stocks were diluted and plated to quantify density through colony counting.

To initiate an experiment, stocks were diluted to target densities determined by the initial community titer (∼1×10^6^ cells) and the number of initial members. For the preliminary synthetic communities, isolates were first combined into 7-member pools, subsequently combined into all 127 combinations of pools, and then distributed into three randomly selected wells containing 600μL of ALM in sterile 1mL deep-well plates. Similarly, for the synthetic communities used to measure interactions, isolates were first combined into desired initial community compositions and then randomly distributed in triplicate into 1mL deep-well plates. All such manipulations were performed under an open atmosphere with a Tecan Freedom Evo liquid handling robot. Deep-well plates were covered with sterilized, loosely fitting plastic lids to allow air exchange. Plates were cultured in the dark at 28°C on high-speed orbital shakers capable of establishing a vortex in the deep-well plates to ensure that the cultures were well-mixed. After 24 hours, 6μL of each culture was manually transferred by multi-channel pipette into new plates containing 594μL of fresh ALM. The new plates were immediately returned to the incubator and the day-old plates were stored at -80°C. The sample plates from the final time point (day 6) were amended with 15% glycerol prior to storage in the freezer to preserve the cultures for subsequent colony counting.

#### DNA extraction

DNA was extracted from synthetic communities using an enzymatic digestion and bead-based purification. Cell lysis began by adding 250μL of lysozyme buffer (TE + 100mM NaCl + 1.4U/μL lysozyme) to 300μL of thawed sample and incubating at room temperature for 30 minutes. Next, 200μL of proteinase K buffer (TE + 100mM NaCl + 2% SDS + 1mg/mL proteinase K) was added. This solution was incubated at 55^o^C for 4 hours and mixed by inversion every 30 minutes. After extraction, the samples were cooled to room temperature before adding 220μL of 5M NaCl to precipitate the SDS. The samples were then centrifuged at 3000 RCF for 5 minutes to pellet the SDS. A Tecan Freedom Evo liquid handler was used to remove 600μL of supernatant. The liquid handler was then used to isolate and purify the DNA using SPRI beads prepared as previously described.^45^ Briefly, samples were incubated with 200μL of SPRI beads for 5 minutes before separation on a magnetic plate, followed by two washes of freshly prepared 70% ethanol. Samples were then resuspended in 50μL ultrapure H2O, incubated for 5 minutes, separated on a magnetic plate, and supernatant was transferred to a clean PCR plate. Purified DNA was quantified using a Picogreen assay (ThermoFisher) and diluted to 0.5ng/μL with the aid of a liquid handler.

#### Sequencing library preparation

Libraries were prepared using Illumina Nextera XT kits and following a custom, scaled down protocol and custom indices (Methods S3). This protocol differed from the published protocol in two ways: 1) the tagmentation reaction was scaled down such that 1μL of purified DNA, diluted to 0.5ng/μL, was added to a solution of 1μL buffer + 0.5μL tagmentase, and 2) a KAPA HiFi PCR kit (Roche) was used to perform the amplification in place of the reagents included in the Nextera XT kit. PCR mastermix (per reaction) was composed of: 3μL 5× buffer, 0.45μL 10mM dNTPs, 1.5μL i5/i7 index adapters, respectively, 0.3μL polymerase, and 5.75μL ultrapure H2O. The PCR protocol was performed as follows: 3 minutes at 72 ^o^; 13 cycles of 95°C for 10 seconds, 55°C for 30 seconds, 72°C for 30 seconds; 5 minutes at 72°C; hold at 10°C. Sequencing libraries were manually purified by adding 15μL of SPRI beads and following the previously described approach, eluting into 12μL of ultrapure H2O. Libraries were quantified by Picogreen assay, and a subset of libraries were run on an Agilent 4200 TapeStation system to confirm that the fragment size distributions were of acceptable quality. The libraries were then diluted to a normalized concentration with the aid of a liquid handler and pooled. The pooled libraries were concentrated on a vacuum concentrator prior to size selection for a 300-600bp range on a Blue Pippin (Sage Science). The distribution of size-selected fragments was measured by TapeStation. Size-selected pool libraries were quantified by Picogreen assay and qPCR (KAPA Library Quantification Kit).

#### Sequencing

We characterized the compositions of our synthetic communities with a shallow metagenomics approach. We chose this approach as opposed to 16S amplicon sequencing as some of our isolates had identical 16S sequences and preliminary work with mock synthetic communities demonstrated that amplicon sequencing yielded less accurate characterizations of community composition. Reference genomes and initial synthetic community samples were sequenced on a HiSeq 4000 platform while follow up synthetic community samples were sequenced on a NovaSeq 6000 platform (paired end 2×150bp for both platforms). Reads were quality filtered and adapter/phiX sequences were removed using BBDuk from the BBTools suite^46^ (v38.81), with the following read quality parameters: qtrim=r, trimq=25, maq=25, minlen=50. Reads were mapped to reference genomes using Seal (BBTools) twice, once with the “ambig” flag set to “toss” (where ambiguously mapped reads were left out) and once with the “ambig” flag set to “random” (where ambiguously mapped reads were randomly distributed to equally likely references). By comparing the results between these two strategies, we identified sets of reference genomes which resulted in high numbers of ambiguous reads due to genomic similarity. We corrected for such ambiguity by reallocating “tossed” reads according to the proportion of unambiguous reads mapped to each isolate in the set for a given sample. To avoid mischaracterizing the composition of our synthetic communities due to contamination or non-specific mapping, for a given sample, isolates with less than 1% of total mapped reads were ignored.

#### Reference genome assembly

Reference genomes for the isolates used in these experiments were assembled using Spades v3.13.0^47^ with the “careful” flag. Assembled genomes were then manually curated in the Anvi’o^48^ software platform (v6.2), specifically using the interactive interface to remove outlier contigs assembled from contaminating sequences. The Anvi’o functions “anvi-summarize” and “anvi-estimate-scg-taxonomy” were also used to estimate the completion and contamination of assembled genomes and assign taxonomy based on single-copy core genes, respectively. The isolate names, taxonomy, and assembly information are presented in Methods S2.

#### Estimating absolute abundance

Absolute density of each community culture was measured by counting colonies from serial dilutions of the cultures. Specifically, glycerol preserved final timepoint samples were plated on 1× tryptic soy agar (TSA) plates, in triplicate serial dilution (3e^-5^, 1e^-6^, and 3e^-6^ dilutions), and cultured at room temperature. Colony forming units (CFU) were counted by eye over the course of a week. Final estimates of absolute abundance were calculated as the mean CFU/μL.

### Quantification and statistical analysis

#### Calculation of interactions

Population level effects were calculated as the ratio of focal isolate density with and without the interactor and presented as (ratio - 1) for ease of interpretation. The per-capita effects were calculated as the change in focal isolate density between contexts with and without the interactor, divided by the density of the interactor from the context in which it was present. To remove spurious interactions that arise from the presence of low abundance isolates close to the 1% relative abundance threshold, we pruned interactions to only include those within a z-score of 1 (±1 SD) from the mean for both the population level and per-capita effect measures, which resulted in the removal of 16% and 12% of each interaction measure, respectively.

#### Statistical analysis and data visualization

Details of statistical tests are reported in the results section and figures. Statistical analysis and figure generation was performed in R^49^ v4.0.2 with aid from the following packages: tidyverse^50^ v1.3.0, reshape2^51^ v1.4.4, and car^52^ v3.0-11. Linear regression was performed in R with the “lm” function and evaluated for adherence to the associated statistical assumptions using the car package. All scripts are provided in the supplementary materials (Methods S1).

## Supplemental information

Document S1. Figures S1–S5 and Tables S2 and S3

Methods S1. R script for processing and analyzing experimental data and making figures, STAR Methods

Methods S2. Table reporting details of the bacterial strains used in this study, STAR Methods

Methods S3: Table containing the custom Illumina library indices used in this study, STAR Methods
